# Blockade of Motor Cortical Long-Term Potentiation Induction by Glutamatergic Dysfunction Causes Abnormal Neurobehavior in an Experimental Subarachnoid Hemorrhage Model

**DOI:** 10.3389/fncir.2021.670189

**Published:** 2021-04-09

**Authors:** Minoru Fujiki, Kazuhiro Kuga, Harushige Ozaki, Yukari Kawasaki, Hirotaka Fudaba

**Affiliations:** ^1^Department of Neurosurgery, School of Medicine, Oita University, Oita, Japan; ^2^Drug Safety Research and Evaluation, Takeda Pharmaceutical Company Limited, Fujisawa, Japan

**Keywords:** electrical stimulation, theta burst stimulation, long-term potentiation, neurobehavior, subarachnoid hemorrhage, motor-evoked potential

## Abstract

Subarachnoid hemorrhage (SAH) is a life-threatening condition that can also lead to permanent paralysis. However, the mechanisms that underlying neurobehavioral deficits after SAH have not been fully elucidated. As theta burst stimulation (TBS) can induce long-term potentiation (LTP) in the motor cortex, we tested its potential as a functional evaluation tool after experimentally induced SAH. Motor cortical inter-neuronal excitability was evaluated in anesthetized rats after 200 Hz-quadripulse TBS (QTS5), 200 Hz-quadripulse stimulation (QPS5), and 400 Hz-octapulse stimulation (OPS2.5). Furthermore, correlation between motor cortical LTP and N-methyl-D-aspartate-receptor activation was evaluated using MK-801, a NMDA-receptor antagonist. We evaluated inhibition-facilitation configurations [interstimulus interval: 3 ms; short-latency intracortical inhibition (SICI) and 11 ms; intracortical facilitation (ICF)] with paired electrical stimulation protocols and the effect of TBS paradigm on continuous recording of motor-evoked potentials (MEPs) for quantitative parameters. SAH and MK-801 completely blocked ICF, while SICI was preserved. QTS5, QPS5, and OPS2.5 facilitated continuous MEPs, persisting for 180 min. Both SAH and MK-801 completely blocked MEP facilitations after QPS5 and OPS2.5, while MEP facilitations after QTS5 were preserved. Significant correlations were found among neurological scores and 3 ms-SICI rates, 11 ms-ICF rates, and MEP facilitation rates after 200 Hz-QTS5, 7 days after SAH (*R*^2^ = 0.6236; *r* = −0.79, *R*^2^ = 0.6053; *r* = −0.77 and *R*^2^ = 0.9071; *r* = 0.95, *p* < 0.05, respectively). Although these findings need to be verified in humans, our study demonstrates that the neurophysiological parameters 3 ms-SICI, 11 ms-ICF, and 200 Hz-QTS5-MEPs may be useful surrogate quantitative biomarkers for assessing inter-neuronal function after SAH.

## Introduction

Survivors of subarachnoid hemorrhage (SAH) are confronted with a variety of long-term problems, including focal neurological deficits, cognitive declines, and higher brain dysfunction, which can be difficult to evaluate (Hackett and Anderson, 2000; Kreiter et al., 2002; Jeon et al., 2010; Sherchan et al., 2011). An objective evaluation of cognitive-neurophysiological function is important for clinical translation, since direct links between quantitative biomarker and clinical symptoms in SAH patients have not been fully established (Kreiter et al., 2002; Sherchan et al., 2011). Furthermore, there are few functional evaluation studies on neurobehavioral and morphological changes following SAH in animals (Thal et al., 2008; Silasi and Colbourne, 2009; Sherchan et al., 2011). Therefore, we intended to perform electrophysiological investigation of cortical excitability, which is potentially related to inter-neuronal functions and might be a possible quantitative biomarker reflecting pathophysiological conditions after SAH.

Theta burst stimulation (TBS) of the hippocampus (3–5 pulses at a frequency of 100 Hz-burst, repeated at 5 Hz) was first shown to facilitate hippocampal-evoked potentials for several hours, on the basis of long-term potentiation (LTP) in animal models (Hess and Donoghue, 1996). TBS stimulation has subsequently been translated to humans using non-invasive transcranial magnetic stimulation (TMS) of the motor cortex (three pulses at a frequency of 50 Hz-burst, repeated at 5 Hz, total, 600 pulses; Huang et al., 2005). It has been used either in intermittent and facilitatory or continuous and inhibitory TBS (iTBS and cTBS, respectively) paradigms for motor-evoked potentials (MEPs) with repetitive TMS (rTMS; Huang et al., 2005). TBS has widespread applications as a non-invasive methodology to evaluate and modify neural-networks within the human brain (Huang et al., 2005; Jung et al., 2016). Therefore, to replicate these human findings, we explored inter-neuronal function in the motor cortex of a rat SAH model after TBS by comparing two standard LTP protocols. Because methodological standardization for LTP induction on the motor cortex remains controversial both in humans and animals, comparison between facilitatory TBS protocol and those for hippocampal LTP induction in experimental condition is informative. Therefore, we designed experimental protocols with a consistent number of total pulses of 1,440 between each condition, at different pulse (200 or 400 Hz) and different burst frequencies [every 200 ms (5 Hz; theta-rhythm), 5 and 10 s]. Hence, total duration of stimulation differed among the TBS and two standard LTP protocol groups (usual durations of each group; 72 s and 30 min, respectively). We also tested the effects of an N-methyl-D-aspartate (NMDA)-receptor antagonist (MK-801) on MEPs after TBS.

## Materials and Methods

### Animals

**Figure 1:**
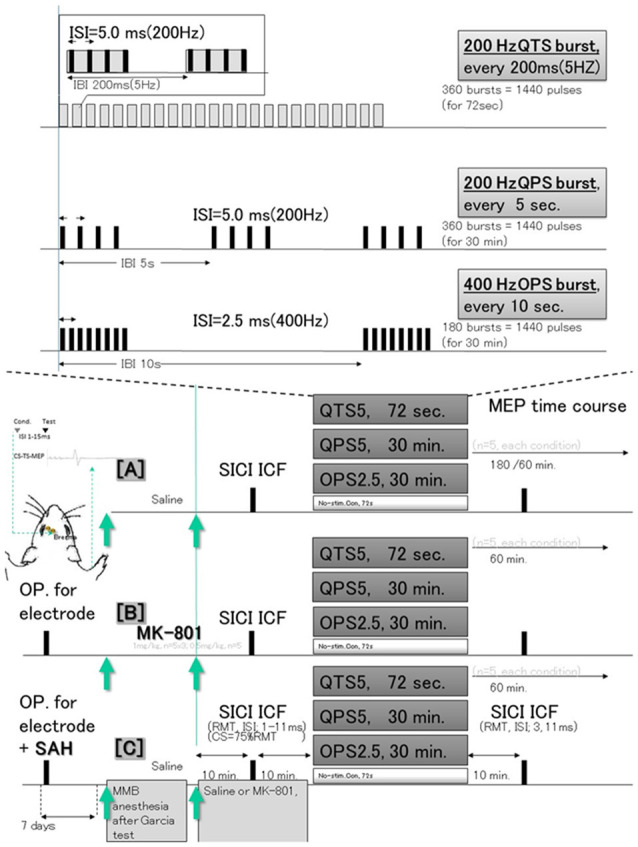
Schematic illustration of the experimental protocol and stimulus configurations. In **(A)**, epidural electrodes [Plastics One with 1.19 mm diameter with a flat tip on two locations: 1.0 mm rostral and 2.0 mm lateral (1R, 2L) and 3.0 mm rostral and 4.0 mm lateral from bregma (3R, 4L); orange circles in **(B)**] were placed 7 days before testing under MMB anesthesia. The screw electrodes were attached in advance to a head connector (Plastics One) such that they were secured with skull screws and dental acrylic for repeated measurements. Motor cortex stimulation for motor-evoked potential (MEP) recording: a train of three biphasic square wave pulses was delivered with an isolated pulse stimulator to achieve temporal summation for selective activation of the motor cortex (0.2 ms per pulse for each polarity; interstimulus interval of 3 ms; refer Fujiki et al., 2020a for detail). QTS5 consists of a burst of four monophasic pulses, 0.2 ms pulse duration at 200 Hz, repeated at 5 Hz, and delivered for 72 s continuously (Jung et al., 2016). QPS5 consisted of the same burst, repeated every 5 s for 30 min, a total 360 bursts based on original reports of QPS of the human motor cortex by Hamada et al. (2008). OPS2.5 consisted of a burst of eight pulses at 400 Hz, repeated every 10 s for 30 min, total 180 bursts, both consisting of 1,440 pulses (Steward et al., 2020). No-stimulation condition was instead produced by unplugging the electrodes at the stimulator while the high frequency stimulation protocol was carried out. Subthreshold CS was set at 75% RMT, while the intensity of TS was adjusted to evoke an MEP of approximately 300 μV (peak-to-peak) in the right BB muscle. Interstimulus intervals (ISIs) of 1, 2, 3, 5, 7, 11, 13, and 15 ms were utilized to test intracortical inhibitions or facilitations. Full ISIs were tested 10 min before QTS5, QPS5 and OPS2.5, while ISIs of 3 and 11 ms were tested 10 min after each stimulation in controls under MMB anesthesia, under MK-801 and 7 days after Subarachnoid Hemorrhage (SAH). Three different experimental sessions [no-Theta Burst Stimulation (TBS) control **(A)**, under MK-801 **(B)**, and 7 days after SAH induction **(C)**] were tested at least 7 days after electrode settings (SAH induction). MMB, medetomidine/midazolam/butorphanol anesthesia (0.15/2.0/5.0 mg/kg, respectively; intraperitoneally. SICI, short latency intracortical inhibition; ICF, intracortical facilitation; QTS5, quadripulse TBS (QTS5 ms; 200 Hz); QPS5, quadripulse stimulation (QPS5; 200 Hz); OPS2.5, octapulse stimulation (OPS2.5; 400 Hz, *n* = 5 each); conditioning stimulus (CS); resting motor threshold (RMT); test stimulus (TS).

All experimental protocols were performed in accordance with the Japanese National Institute of Health Guide for the Care and Use of Laboratory Animals and approved by the Oita University Ethical Review Committee (protocol number 192302). Following a sample size calculation, we conducted our experiments on a total of 50 adult, male Sprague–Dawley rats (body weight, 295–377 g, aged 8.2 ± 0.2 weeks old) that were housed in a controlled environment with room temperature (24.5–25.0°C), 12/12-h light/dark cycle, and constant humidity. Rat food pellets and tap water were provided *ad libitum* between experimental procedures. The study was composed of three different experimental sessions involving 50 animals: [A] continuous MEP recording after 200 Hz-quadripulse TBS, 200 Hz-QPS, and 400 Hz-OPS (QTS5, QPS5, and OPS2.5, respectively, *n* = 5, each condition); [B] the same procedure as in [A], but with NMDA-receptor antagonist MK-810 (1 mg/kg) administration to explore the correlation between MEP modification and NMDA activation (*n* = 5, each condition); [C] same procedure as in [A], but 7 days after SAH to explore the underlying functional consequences and pathophysiology (*n* = 5, each condition; Figure 1).

### Induction of Cisterna Magna SAH Model

Seven days before testing, the rats were deeply intraperitoneally anesthetized with a combination of medetomidine/midazolam/butorphanol anesthesia (0.15/2.0/5.0 mg/kg, respectively). The anesthetic conditions were continuously verified through the experiment (maintained during electrode implantation, SAH induction and during data recording 7 days after) in all animals (Fujiki et al., 2004; Fujiki et al., 2020a,b; Qin et al., 2015; Mishra et al., 2017). Briefly, the absence of pedal withdrawal (“toe-pinch”) reflex was used for assessment of anesthesia and analgesia depth, and the body temperature was maintained at 37°C intra- and postoperatively with a temperature-controlled heating pad.

Autologousblood (300 μl) was injected into the subarachnoid space over a 3-min time frame from a catheter within the cisterna magna. The injection was repeated 48 h after first injection (Qin et al., 2015).

### Continuous Recording of MEPs

MEPs were continuously recorded from the contralateral right biceps brachii muscle *via* wire-electrodes, as per previously described methods (Fujiki et al., 2004; Fujiki et al., 2020a,b; Mishra et al., 2017). Electrode setting and base-MEP recordings were performed before the induction of SAH. During MEP measurements, rats were anesthetized with amedetomidine/midazolam/butorphanol combination to preserve the motor responses in the same manner with SAH induction.

### Paired Motor Cortex Electrical Stimulation for Inhibition-Facilitation Configurations

Intracortical inhibition or facilitation [short-latency intracortical inhibition (SICI) and intracortical facilitation (ICF)] were tested using a paired electrical and subthreshold conditioning stimulus (CS) preceding a suprathreshold test stimulus (TS) after resting motor threshold (RMT) determination (Kujirai et al., 1993; Rothwell, 1997; Fujiki et al., 2020a). The RMT was measured by varying the stimulator output by 0.1-mA-step until six stable peak-to-peak-50 μV-MEP elicitations were obtained for every 12 trials. Two isolated stimulators connected with acustom-made single-stimulus-electrode switching unit were controlled for appropriate stimulus intervals (1, 2, 3, 5, 7, 11, 13, and 15 ms) and intensity (0.5–1.2 mA) in a similar manner as paired TMS (Vahabzadeh-Hagh et al., 2011; Hsieh et al., 2012; Fujiki et al., 2020a).

### Motor Cortex Electrical Stimulation: 200 Hz-Quadripulse TBS, 200 Hz-Quadripulse and 400 Hz-Octapulse Stimulation for LTP Induction

A 200 Hz-quadripulse TBS (QTS5 ms), 200 Hz-quadripulse stimulation (QPS5), 400 Hz-Octapulse stimulation (OPS2.5; *n* = 5 each) or an absent (no-) stimulation was applied for 72 s. Three different experimental sessions (no-TBS [A], treated with MK-801[B] and 7 days after SAH induction [C]) were tested for at least 7 days after electrode settings (SAH induction; Figure 1).

The animals were humanely sacrificed after data collection with an overdose of anesthetic prior to decapitation. The brains were then prepared for verification of electrode position and histological evaluation after SAH. High-frequency stimulation (QTS5, QPS5, and OPS2.5) was delivered at an intensity of 75%-RMT (0.5–1.2 mA; Yang et al., 2019; Fujiki et al., 2020a) for 1,440 pulses and post stimulus-continuous MEPs were recorded at 0.1 Hz for 60 or 180 min. The no-stimulation condition was produced by unplugging the electrodes while the high-frequency stimulation protocol was carried out.

### Effects of MK-801

A separate group of animals were prepared to explore the correlation between motor cortical LTP induction after QTS5, QPS5, and OPS2.5 and NMDA-receptor activation, with rats receiving an NMDA-receptor antagonist, MK-801 (1 mg/kg, intraperitoneally; FUJIFILM Wako Pure Chemical Corporation, Japan; [B] in Figure 1, *n* = 15). Low-dose MK-801 (0.5 mg/kg) was tested only for MEPs and CS preceding TS-MEP recording (*n* = 5). Based on the previously reported pharmacokinetic and pharmacodynamic properties of MK-801 in rats, we adjusted motor cortical LTP induction periods to make them comparable with the bet time window (Wegener et al., 2011). Furthermore, to confirm whether MK-801 was at a steady state during the induction of motor cortical LTP by QTS5, QPS5, and OPS2.5, the pharmacodynamics of MK-801 were tested by means of functional observation battery (FOB) using eight separate group of animals. We verified the positive steady symptoms during 1-h-post-dose periods (see Supplementary Material for detail). Thus, we could confirm that MK-801 doses were steady during the induction of LTP, but interaction with anesthetic combination was not confirmed.

### Neurological and Histological Evaluation After Experimental SAH

The SAH grading of animals at 7 days was determined according to previously described methods (Sugawara et al., 2008). Modified Garcia’s neurological scores (consisting of six tests; scored 3–18) were evaluated at 7 days after SAH by a blinded investigator (Garcia et al., 1995). Animals that survived for 7 days after SAH were evaluated for histological analysis (hematoxylin and eosin and silver stain) immediately after MEP evaluations (Fujiki et al., 2004).

### Data Analysis

The MEP data were analyzed offline, as per previous reports (Sykes et al., 2016; Fujiki et al., 2020a). MEP amplitudes were measured in a peak-to-peak manner, with 120% RMT stimulus intensity used for MEP recording with a 10-s interval, six individual sweeps run each minute (except for the initial 30 min in which a 10-s interval, three sweeps protocol was run every 5 min). This allowed us to record MEP development during QPS5 and OPS2.5. Amplitude was normalized to the final 5-min baseline amplitude and expressed as percentage-change, allowing for between-subject comparisons and grouping into 5 min bins.

Different groups of animals were compared using analysis of variance (ANOVA) with a Student–Newman–Keul *post-hoc* analysis (SPSS, Cary, NC, USA). Data are presented as mean ± standard error of the mean. For QTS5, QPS5, and OPS2.5 effects, statistical significance of group differences was analyzed by ANOVA with time (TIME) as within-subject factor and group (GROUP) as between-subjects factor. This was followed by *post-hoc* Holm test. To investigate whether the time effect differed among groups, we confirmed the TIME × GROUP interaction. Differences were considered significant at *P* ≤ 0.05.

## Results

### General Condition and Histopathology 7 Days After SAH

**Figure 2:**
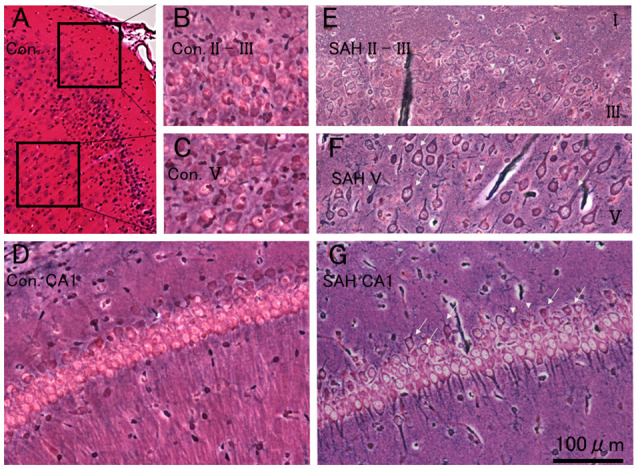
Histopathological results at 7 days after SAH. Photomicrograph indicating neuronal density in the motor cortical layers I-III, V, and CA1 region of the hippocampus in a rat 7 days after no-TBS control **(A,B,C,D)** and SAH **(E,F,G)**. Note that the prominent cell damages in the motor cortical layers III and V area in the rat subjected to SAH [showing transition between healthy cells (arrows) and with dead or dying cells (arrowheads)] and the relative absence of neuron loss in the hippocampal CA1 area (showing a mixture of some dying cells in healthy cells). Panel **(C)** illustrates cell density in a control case. Bars = 200 μm **(A)** and 100 μm (**B,C,D,E,F** and **G**). Areas in panels **(B,C)** correspond to the inset area in Panel **(A)**.

Physiological parameters were within the normal range before induction of SAH [pH, 7.45 ± 0.01; pO_2_ (mmHg), 75.2 ± 1.8; pCO_2_ (mmHg), 47.1 ± 2.1; and Hematocrit (%), 51 ± 0.8]. All control MEPs were abolished immediately after SAH induction on the day which became nil. There was no mortality in either no-TBSor SAH animals during the 7 days. The average SAH grade was 3.2 ± 1.1. Figure 2 illustrates the characteristic pattern of neuronal morphology in the rat motor cortical layers I-III, V, and CA1 region of the hippocampus after 7 days in the no-TBS control (A, B, C, D) and SAH (E, F, G) conditions.

As previously documented (Ostrowski et al., 2005), there was extensive neuronal damage, predominantly in motor cortical layers III and V area in rats, subjected to SAH (compare Figures 2B,C controls with Figures 2E,F). Notably, the neurons in the hippocampal CA1 area were relatively preserved. Moreover, rather than having drastic cell loss, rats subjected to SAH displayed morphological abnormalities in their cortical neurons. However, whether there were any quantitative differences in cell number between the SAH and no-TBS control animals were not investigated.

### MEP Basic Waveforms

**Figure 3:**
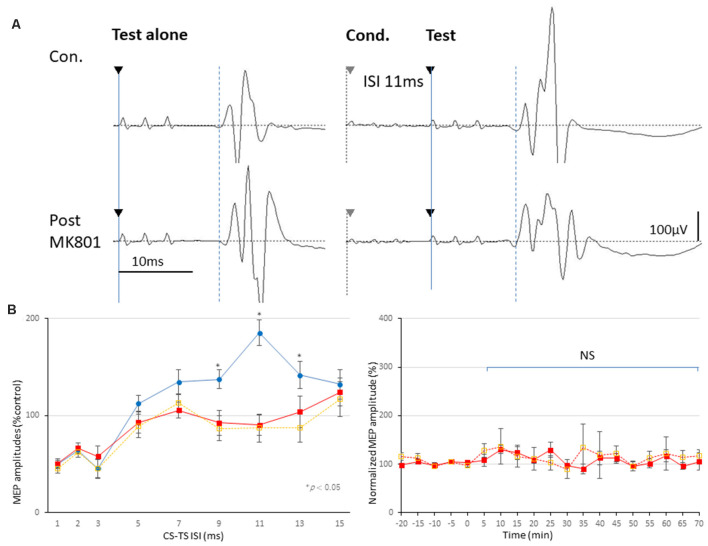
Effects of MK-801 on MEPs and 3 ms-inhibition and 11 ms-facilitation patterns. **(A,B)** Basic waveform of MEPs recorded from the biceps muscle is composed of short-latency (approximately 14 ms) biphasic waves. Inhibition and facilitation patterns of MEPs at ISIs of 3 and 11 ms under medetomidine/midazolam/butorphanol (MMB) anesthesia (**A,B**; Circle). This phenomenon is reminiscent of the SICI and ICF in the human motor cortex using paired pulse TMS. Rats with MK-801 injection (1 mg and 0.5 mg/kg, i.p.) demonstrated significant suppression at 11 ms-facilitation but consistent amplitudes of test MEPs. Analysis of Variance (ANOVA) revealed that the values were significantly smaller in the MK-801 groups at an ISI of 11 ms. *P < 0.05 (**B**; square, solid and dotted line, respectively). Note that an absence of statistically significant effects of MK-801 administrations on parameters RMT, latency, or amplitude was found **(B)**. NS, statistically not significant.

The final 5 min of MEP baseline parameters, including RMT, latency, and amplitudes, in no-TBS controls, MK-801, and SAH groups are summarized in Table 1. No qualitative differences were found among the groups. In addition, there was no statistically significant effect of MK-801 administration and SAH on RMT, latency, or amplitude (Figures 3A,B).

**Table 1:** MEP baseline parameters.

		Control (*n* = 15)	MK-801 (*n* = 15)	SAH (*n* = 15)	*F*	*P*
Baseline	RMT (mA)	1.05 ± 0.04	1.04 ± 0.04	1.04 ± 0.03	*F*_(2,42)_ = 0.035	0.97
	latency (ms)	14.2 ± 0.31	13.6 ± 0.32	13.5 ± 0.34	*F*_(2,42)_ = 1.466	0.24
	amplitude (μV)	280 ± 9.72	291 ± 18.64	312 ± 9.18	*F*_(2,42)_ = 1.485	0.24

*MEP, motor evoked potential; RMT, resting motor threshold; SAH, subarachnoid hemorrhage*.

### Inhibition-Facilitation Configurations With Paired-Stimulation Protocol-MEPs

Paired-stimulation protocol-MEPs showed inhibition (ISIs, 1–3 ms; SICI) and facilitation (ISI, 11 ms; ICF) patterns (left graph, Figure 3B). While MK-801 completely blocked 11 ms-ICF, 3 ms-SICI was preserved [low dose (0.5 mg/kg); orange-dotted lines and high dose (1.0 mg/kg); red-lines, respectively; asterisk (*) indicate significance; *P* < 0.05; Figures 3A,B]. One-way ANOVA revealed that significant difference at an ISI of 9 (*F*_(1,28)_ = 16.36, *P* = 0.00037), 11 (*F*_(1,28)_ = 45.51, *P* < 0.0001), 13 (*F*_(1,28)_ = 7.86, *P* = 0.0096; 1 mg/kg), and ISI of 9 (*F*_(1,18)_ = 8.69, *P* = 0.0082), 11 (*F*_(1,18)_ = 18.62, *P* = 0.00037), and 13 ms (*F*_(1,18)_ = 5.05, *P* = 0.038; 0.5 mg/kg), respectively.

### LTP of MEPs After 200 Hz-QTS5 in No-TBS-Control Rats

**Figure 4:**
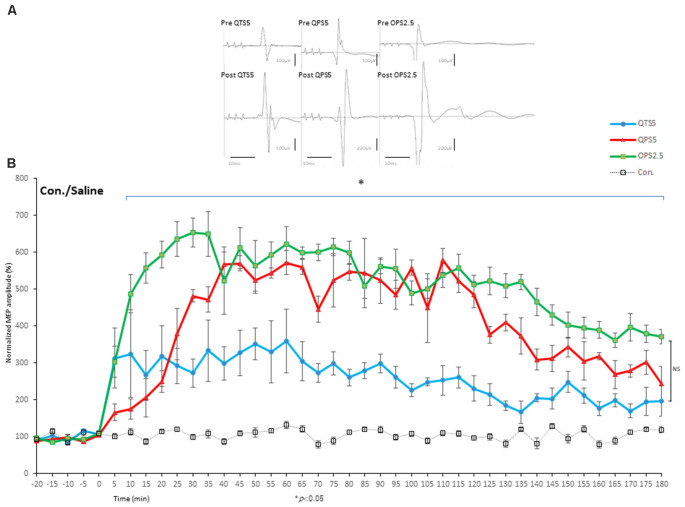
Quantitative evaluation of the MEPs after 200 and 400 Hz high-frequency stimulation in control animals. Time course of MEPs after QTS5, QPS5, and OPS2.5 in no-TBS controls. MEP facilitation after OTS5, QPS5, and OPS2.5 stimulation in control animals **(A,B)**. Note that 10 ms of horizontal calibration bar and 100 μV vertical bar in pre-stimulation and QTS5, whereas 200 μV vertical bar in post-QPS5 and OPS2.5. MEPs were strongly facilitated immediately after motor cortical QTS5 (blue-line), lasting upto 180 min under MMB anesthesia. Both QPS5 and OPS2.5 also facilitated MEPs lasting up to 180 min under MMB anesthesia (red- and green-line, respectively). ANOVA revealed a significant main effect of group on MEP, whereby the effects of stimulation differed among the four groups (main effect of GROUP, *F*_(3,55)_ = 148.23, *P* < 0.001; main effect of TIME, *F*_(13,224)_ = 18.56, *P* < 0.001; interaction of GROUP × TIME, *F*_(39,224)_ = 4.55, *P* < 0.001). A *post-hoc* analysis indicated significant increases, compared to the no-TBS group, in the MEP amplitudes after the stimulation in the QTS5, QPS5, and OPS2.5 groups (*P* < 0.001). Multiple comparisons between the QTS5 and no-TBS groups were conducted at each time point. Our results indicated the MEP amplitudes in the QTS5 group were significantly increased compared with those in the no-TBS group at several time points (*P* < 0.001, respectively). Differences in the increase of MEP amplitudes were observed immediately following stimulation and persisted for more than 3 h (data not shown) following stimulation, suggesting persistent TBS effects on the MEP amplitudes. Both QPS5 and OPS2.5 appeared to induce stronger facilitation than QTS5 during stimulation for the first 30 min (statistically not significant), but there were no significant differences between the three groups in the final 30 min (*P* > 0.05). NS, statistically not significant. *P < 0.05.

**Figure 5:**
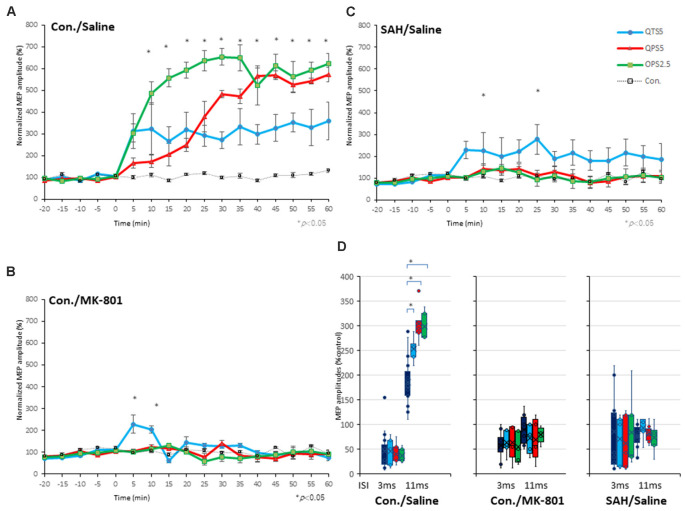
Quantitative evaluation of the MEPs after 200 Hz, 400 Hz high-frequency stimulation in control, MK-801 treated, and SAH animals. Time course of MEPs after QTS5, QPS5, and OPS2.5 in no-TBS controls, animals under MK-801, and SAH models. **(A)** MEP amplitudes in the QTS5 (blue-line), QPS5 (red-line), and OPS2.5 (green-line) groups were significantly increased compared with those in the no-TBS group at several time points. **(B)** MEP amplitudes in the QTS5 under MK-801 group were significantly increased compared with those in the no-TBS group at transient time points (5 min and 10 min post-stimulation, *P < 0.05). **(C)** MEP amplitudes in the QTS5 after SAH group were increased compared with those in the no-TBS group at several time points (10 min and 25 min post-stimulation, *P < 0.05). Note an absence of statistically significant effects of SAH on parametersRMT, latency, or amplitude was found. **(D)** ICF at ISI of 11 ms were significantly facilitated after QTS5, QPS5, and OPS2.5, whereas SICI at an ISI of 3 ms was not affected under MMB anesthesia in controls (left graph). Facilitations after stimulation in MMB controls were suppressed both under MK-801 and 7 days after SAH (middle and right graph).

Motor cortical QTS5 continuously facilitated MEPs immediately after stimulation (Figure 4A), lasting for 180 min (*P* < 0.05; Figure 4B). Both QPS5 and OPS2.5 facilitated MEPs during 30 min stimulation periods, also lasting 180 min (*P* < 0.05; Figures 4A,B). ANOVA revealed that significant main effect of group on MEP in normalized MEP amplitude over time, while *post-hoc* comparison indicated that MEP amplitudes were significantly higher than those in the no-TBS group (*P* < 0.001; Figure 4). QTS5, QPS5, and OPS2.5 significantly facilitated 11 ms-ICF (QTS5: *F*_(1,18)_ = 7.99, *P* = 0.011; QPS5: *F*_(1,18)_ = 22.08, *P* = 0.00018; OPS2.5: *F*_(1,18)_ = 21.51, *P* = 0.0002 < 0.05; values between groups: *F*_(2,13)_ = 3.47, *P* = 0.65; Figure 5D, left graph), whereas 3 ms-SICI was not affected in controls.

### SICI, ICF, and 200 Hz-QTS5 Induced TLP Effect on SAH-Rat MEPs

**Figure 6:**
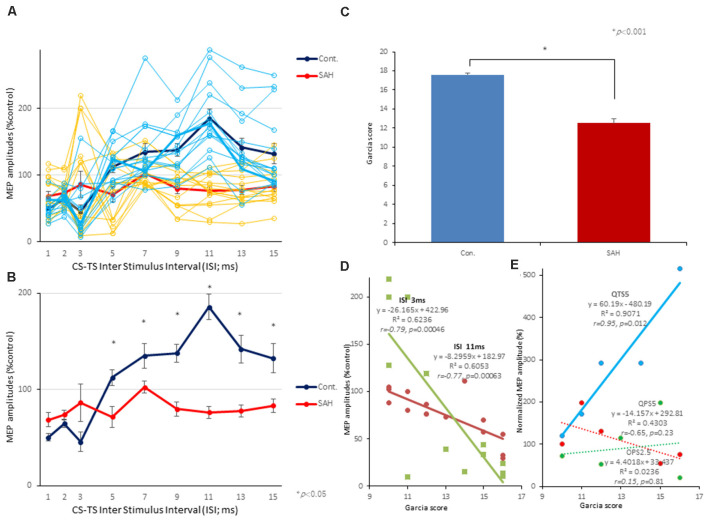
Effects of SAH on 3 ms-inhibition (SICI) and 11 ms-facilitation (ICF) patterns, neurological scores, and correlations between parameters for SICI/ICF and MEP amplitudes after 200 Hz-QTS5. Individual profiles of normalized MEPs in each ISI in controls and SAH animals under MMB anesthesia **(A,B)**. Averaged normalized MEPs under SAH (red) and control (blue) at CS at 75% RMT preceding TS-MEPs **(A,B)**. No-TBS control-MMB-anesthetized rats demonstrated the significant inhibition at ISIs of 3 ms and facilitation at 11 ms, whereas SAH rats demonstrated the significant suppression at 11 ms-facilitation at 75% RMT-CS. Inhibition at an ISI of 3 ms were slightly preserved (statistically not significant; **A,B**). ANOVA revealed that the values were significantly greater in the control group than in the SAH group at an ISI of 11 ms. *P < 0.05. Neurological scores of rats after SAH were significantly lower than those of the control group at 7 days after onset (*P* < 0.001; **C**). There were significant correlations between Garcia’s neurological scores and 3 ms-inhibition rates (light green solid line) and 11 ms-facilitation rates (brown solid line; **D**). There were significant correlations between Garcia’s neurological scores and normalized MEP amplitudes at 25 min, only after 200 Hz-QTS5 (*P* = 0.012; blue solid line) but not after QPS5 (*P* = 0.23; red dashed line) and OPS2.5 (*P* = 0.81; green dashed line; **E**).

Both 3 ms-inhibition and 11 ms-facilitation configurations were suppressed after SAH (*P* < 0.05; Figures 6A,B). One-way ANOVA revealed a significant difference at an ISI of 5 (*F*_(1,28)_: 8.740, *P* = 0.0062) 7 (*F*_(1,28)_: 5.43, *P* = 0.027), 9 (*F*_(1,28)_: 23.10, *P* < 0.0001), 11 (*F*_(1,28)_ = 56.02, *P* < 0.0001), 13 (*F*_(1,28)_ = 17.25, *P* < 0.001), 15 (*F*_(1,28)_ = 8.64, *P* = 0.0065) ms, respectively. There was a tendency for the 3 ms-SICI to be suppressed, although the change was not statically significant (Figure 6B). Therefore, intracortical inhibition and facilitation profiles 7 days after SAH in this study, were entirely different than normal controls.

It should be noted that CS preceding TS-MEP amplitudes (% control) at both 3 ms-inhibitory and 11 ms-facilitatory phases were suppressed approximately 25% compared to TS-alone base-MEP amplitudes (Figure 6B; red-line). All MEP facilitations after QPS5, OPS2.5-stimulations were completely suppressed, while those after QTS5 were preserved both with MK-801 and 7 days after SAH (compare Figures 5B,C with Figure 5A).

ANOVA revealed that significant main effect of group on MEP after QTS5 under MK-801, whereby the effects of stimulation differed among the four groups (main effect of GROUP, *F*_(3,55)_ = 8.42, *P* < 0.001; main effect of TIME, *F*_(13,224)_ = 4.52, *P* < 0.001; interaction of GROUP × TIME, *F*_(39,224)_ = 3.07, *P* < 0.001; Figure 5B).

A *post-hoc* analysis indicated that significant main effect group increases in the MEP amplitudes compared to those in the no-TBS group, after stimulation in the QTS5 and MK-801 groups (*P* = 0.015). Multiple comparisons between the QTS5 and no-TBS groups were conducted at each time point. Our results indicated that the MEP amplitudes in the QTS5 group were significantly higher than those in the (no-TBS) stimulation control sham group at transient initial time points (5, 10 min following stimulation:* P* = 0.01, respectively; compare Figure 5B with Figure 5A).

ANOVA revealed that significant main effect of group on MEP after QTS5 7 days after SAH, whereby the effects of stimulation differed among the four groups (main effect of GROUP, *F*_(3,55)_ = 24.37, *P* < 0.001; main effect of TIME, *F*_(13,224)_ = 0.858, *P* = 0.5979; interaction of GROUP × TIME, *F*_(39,224)_ = 0.57, *P* = 0.98; Figure 5C).

A *post-hoc* analysis indicated that significant main effect group increases in the MEP amplitudes compared to those in the no-TBS group, after the stimulation in the QTS5 and SAH group (*P* < 0.001). Multiple comparisons between the QTS5 and no-TBS groups were conducted at each time point. Our results indicated that the MEP amplitudes in the QTS5 group were significantly higher than those in the no-TBS group at two time points (10, 25 min following stimulation: *P* = 0.048, 0.044, respectively; compare Figure 5C with Figure 5A).

All 11 ms-ICF facilitations after QTS5, QPS5, and OPS2.5 stimulations were suppressed both under MK-801 and 7 days after SAH (Figure 5D, middle and right graph).

### Correlation Between Neurological Score and 3 ms-SICI, 11 ms-ICF, and 200 Hz-QTS5-MEPs After SAH

The mean neurological scores for the control and SAH groups are compared in Figure 6C (statistical significance was found at 7 days after SAH onset; *P* < 0.001). Garcia’s neurological scores and 3 ms-SICI rates and 11 ms-ICF rates were significantly correlated (*R*^2^ = 0.6236; *r* = −0.79; *P* = 0.00046 and *R*^2^ = 0.6053; *r* = −0.77; *P* = 0.00063, respectively; Figure 6D).

No statistical difference was found in the neurological scores among pre-QTS5, pre-QPS5, and pre-OPS2.5 groups (scores between groups; *F*_(2, 12)_ = 0.077, *P* = 0.93; 12.6 ± 1.08, 12.8 ± 1.15, 13.2 ± 1.07, respectively).

Garcia’s neurological scores and normalized MEP amplitudes at 25 min, only after QTS5, were significantly correlated (*R*^2^ = 0.9071; *r* = 0.95; *P* = 0.012; blue solid line in Figure 6E), but not after QPS5 and OPS2.5 (*R*^2^ = 0.4303; *r* = −0.65; *P* = 0.23; red dashed line and *R*^2^ = 0.0236; *r* = 0.15; *P* = 0.81; green dashed line, respectively; Figure 6E).

## Discussion

We verified the pattern of MEP facilitation, as a consequence of three different LTP-inducing, high-frequency electrical stimulations of the motor cortex in control and SAH model rats. For our study, we used the same pattern of stimulation for MEP facilitation in the motor cortex as shown in previous human and animal studies (QTS5; Jung et al., 2016, QPS5; Hamada et al., 2008, OPS2.5; Steward et al., 2020, respectively). Four or eight pulses at high frequency (200 or 400 Hz-burst), repeated at 5 Hz (four pulses at 5 ms inter-pulse interval; QTS5), every 5 s (four pulses at 5 ms inter-pulse interval; QPS5) or every 10 s (eight pulses at 2.5 ms inter-pulse interval; OPS2.5), under a constant number of 1,440 pulses, were consistent between each condition.

Using continuous MEP recording, we found that high-frequency repetitive electrical stimulation at 200 and 400 Hz strongly activated MEPs, persisting for 180 min under anesthesia. SAH and MK-801, the NMDA-receptor antagonist, completely blocked MEP facilitation, while it was preserved after QTS5 treatment.

### Correlation Between Morphological and Neurophysiological Changes After SAH

Morphological damages following SAH, demonstrating substantial neuronal damages in the motor cortical layers III and V were similar to those reported in previous study (Ostrowski et al., 2005). Careful correlational interpretation should be done between morphological changes and present neurophysiological results. The final output neuronal cells in layer V contributing to generate MEPs are affected both by corticospinal neurons and by surrounding GABAergic and glutamatergic interneurons, which connect with corticospinal neurons. Thus, our results are based on cortical excitability and potentially summarize the overall inter-neuronal functions.

### SICI, ICF, and Motor Cortical LTP for Surrogate Quantitative Biomarker for SAH

The three neurophysiological parameters (3 ms-SICI, 11 ms-ICF, and 200 Hz-QTS5-MEPs) that were found to correlate with behavioral scores could be useful as surrogate quantitative biomarkers. However, we did not use direct measurements and therefore these findings do not reflect the degree of GABAergic and glutamatergic deficiency and/or plasticity 7 days after SAH.

We attempted to determine whether CS preceding 3 ms-SICI, 11 ms-ICF, and LTP-like MEP responses after high-frequency stimulations could provide an accurate correlation with the clinical symptoms observed in patients with SAH.

### 200 Hz-QTS of the Rat Motor Cortex Facilitates MEP; Rationale for Glutamatergic Mechanisms

Considering the difference between electrical stimulation and TMS (while current density close to the electrodes is higher than that in between electrodes, it is more uniform with TMS), stimulus parameters of electrical stimulation as in TMS may not always induce the same neuroplasticity. Furthermore, stimulus parameters for experimental hippocampal LTP induction have never been applied for motor cortical LTP induction both in human and animals. To understand the underlying mechanisms and verify compatibility with human and animal results, experiments comparing with other patterns of stimulation will be informative. The fact that both QPS5 and OPS2.5 appeared to induce stronger facilitation than QTS5 during stimulation for the first 30 min on control animals, but there were no significant differences among the three groups in the final 30 min, is of interest. Longer stimulus duration providing stronger developing phases in QPS5 and OPS2.5 (QTS5; 72 s vs. 30 min) is one possibility (Nakamura et al., 2016). Future studies in this regard are warranted.

The present results re-confirmed 11 ms-ICF as glutamatergic inter-neuronal hypothesis within the motor cortex, depending on NMDA-receptor activation both in humans and rats (Di Lazzaro and Rothwell, 2014; Di Lazzaro et al., 2018; Fujiki et al., 2020a). Indeed, the facilitatory effect of QPS5 is proposed to be glutaminergic receptor-mediated, because ICF is facilitated after TMS-QPS5 in the human motor cortex (Hamada et al., 2008). Furthermore, a 200 Hz-burst pattern (ISI of 5 ms) is considered optimal for inducing sufficient-maximal postsynaptic Ca^2+^ influx, triggering intra- and/or inter-neuronal signaling cascades for the LTP induction *via* activity dependent NMDA-receptor activation (Kenney and Manahan-Vaughan, 2013). The OPS2.5 facilitatory effect that produces a 400 Hz-burst (ISI of 2.5 ms) has been considered a glutaminergic receptor-mediatedgold standard in the animal hippocampal LTP induction model, which has been recently applied a motor cortical electrical stimulation induced-LTP model (Steward et al., 2020).

Transient MEP facilitation after QTS5 under MK-801 suggests different underlying mechanisms between QPS5, OPS2.5, and QTS5. The importance of an “inter-burst interval of 5 Hz in TBS” has been proposed, with GABAergic inhibition in intracortical interneurons preventing the decay in burst efficiency and facilitating long-term MEP and hippocampal LTP (Kenney and Manahan-Vaughan, 2013; Jung et al., 2016). All these observations in relation to NMDA receptor-dependent synaptic plasticity are distinctly correlated with frequency-dependency (Kenney and Manahan-Vaughan, 2013; Jung et al., 2016; Fujiki et al., 2020b). Although there is evidence that LTP-like MEP facilitation after high-frequency stimulation of the motor cortex, which may be the part of the common mechanisms for hippocampal LTP induction (Kelleher et al., 2004), 3 ms-SICI, 11 ms-ICF, and 200 Hz-QTS5-MEPs are more broadly applicable, as sensitive-quantitative surrogate markers of NMDA-receptor activation that underly synaptic plasticity after SAH.

Our results expand the current consensus of inter-neuronal profiles affected by high-frequency stimulation (200 Hz-TBS, 200 and 400 Hz) of the rat hippocampus to the motor cortex in the experimental settings. We further demonstrated that these stimulation paradigms activate NMDA receptors, and that NMDA-receptor mediated glutamatergic inter-neuronal dysfunction correlates with neurobehavioral deficits after SAH. However, these results need to be verified in humans.

### Limitations and Future Work

First, considering that the animals in the present study were under stable anesthetic conditions, it should be noted that RMT and all altered parameters in MEPs, SICIs, ICFs, and MEP facilitations after LTP-inducing high-frequency stimulations through the experiments may have been affected by midazolam, a GABA-A agonist-based anesthetic combination. However, in contrast to urethane, a compound commonly used for synaptic plasticity studies for non-survival single session (Sykes et al., 2016), the present combination (applicable for repeated survival experiments) is also favorable for multi-synaptic MEP studies (Fujiki et al., 2020a). Second, the relationship between pathophysiological cascades of SAH and NMDA glutamatergic inter-neuronal dysfunction remains unclear. In this regard, further careful and detailed studies are required to elucidate the links between neuro-biological effects at the molecular level to address safety issues with stimulation of the motor cortex, as well as high-frequency stimulation, and induction of LTP in the human brain. This is consistent with the current consensus that even non-invasive-TMS in both humans and animals can induce LTP/LTD and alter synaptic plasticity with cellular and/or molecular mechanisms (Carmel et al., 2010; Müller-Dahlhaus and Vlachos, 2013; Rodger and Sherrard, 2015; Fujiki et al., 2020a,b). Therefore, non-invasive-brain stimulation-based therapies may be beneficial for treating neurobehavioral deficits after SAH.

## Conclusion

QTS5, QPS5, and OPS2.5 strongly facilitated MEPs. MK-801 and SAH completely blocked these MEP facilitations, while MEP facilitation after QTS5 was preserved. Garcia’s neurological scores and 3 ms-SICI rates, 11 ms-ICF rates, and MEP facilitations after 200 Hz-QTS5-MEPs were significantly correlated. Neurophysiological evaluation of 3 ms-SICI, 11 ms-ICF, and 200 Hz-QTS5-MEPs may be useful surrogate quantitative biomarkers for inter-neuronal functional evaluation after SAH.

## Data Availability Statement

The raw data supporting the conclusions of this article will be made available by the authors, without undue reservation.

## Ethics Statement

The animal study was reviewed and approved by Oita University Ethical Review Committee.

## Author Contributions

MF, KK, HO, and YK designed the research and wrote the article. MF, HF, and YK performed experiments. MF, KK, HO, and HF analyzed data. All authors contributed to the article and approved the submitted version.

## Conflict of Interest

KK and HO were employed by the Takeda Pharmaceutical Company Limited. The remaining authors declare that the research was conducted in the absence of any commercial or financial relationships that could be construed as a potential conflict of interest.
