# Supplementary material for: Blockade of Motor Cortical Long-Term Potentiation Induction by Glutamatergic Dysfunction Causes Abnormal Neurobehavior in an Experimental Subarachnoid Hemorrhage Model
Source: Front Neural Circuits. 2021 Apr 9;15:670189. doi: 10.3389/fncir.2021.670189 (PMC8063030; doi:10.3389/fncir.2021.670189)
Supplement: Supplementary file 1 [file Data_Sheet_1.docx]

**Suppl. Materials and Methods-*Effects of MK-801*. Data on pharmacodynamics of MK-801**

[**2. Materials and Methods**; *2.6. Effects of MK-801*]

[Main description]

On the basis of previously reported pharmacokinetic and pharmacodynamic properties of MK-801 in rats, we adjusted the motor cortical LTP induction periods so they were comparable with the bet time window (Wagener et. al., 2011). Furthermore, to confirm whether MK-801 was at a steady state during the induction of the motor cortical-LTP by QTS5, QPS5, and OPS2.5, the pharmacodynamics of MK-801 were tested by means of functional observation battery (FOB) using 8 separate set of animals. We verified positive steady symptoms during 1-hour-post-dose periods.

The following findings were noted at 1-hour-post-dose at ≥0.5 mg/kg: a weak muscular tone of handling, slight flaccidity, abnormal gait (staggering gait at 0.5 mg/kg, and leg dragging at 1 mg/kg), left circling, head shaking, no approach response, no/weak righting reflex, weak response proprioception, loss of forelimb strength, no visual placing response, abnormal wheelbarrowing, and abnormal hopping reaction (both forelimb and hindlimb).

The following findings were noted at 1-hour-post-dose at 1 mg/kg: prone position, twitch, salivation, a decrease in locomotor activity, and weak click response (pinna reflex). In addition, an increase in locomotor activity was noted at ≥0.5 mg/kg at 6-hours-post-dose. A statistically significant increase in hindlimb landing foot-splay was noted at 6-hours-post-dose at ≥0.5 mg/kg. No statistically significant differences from the control group were noted in body temperature and pupil size at any time point at any dose.

Thus, MK-801 doses were steady during induction of LTP, but interaction with anesthetic combination was not confirmed.

[Examinations and Methods]

The first day of the dosing period was defined as Day 1. The following examinations (clinical signs, body weights, FOB) were performed during the experimental period. The data for clinical signs during the pre-dose period and body weights are not appended to this final report because the data were used only for animal selection, group allocation, and calculation of the dose volume.

[FOB]

The parameters listed below were examined for each animal in the following order: home cage observations, handheld observations, open-field observations, and observations of sensory function and reaction. The observations were conducted at pre-dose and 1 and 6 hours post-dose. Each animal and its housing cage were transferred to another rack after each observation on the day of dosing. The position of the housing cage on the different rack corresponded to where the animal was located on the original rack before the transfer. The FOB was performed on 3 different experimental days.

Home cage observations: Piloerection, skin color, body position, respiration, twitch, convulsion, defecation, and urination

Handheld observations: Ease of handling, body (rectal) temperature, body tone, exophthalmos, lacrimation, salivation, and pupil size

Open-field observations: Abnormal gait, tremor, bizarre behavior, palpebral closure, and locomotor activity

Observations of sensory function and reaction: Click response, approach response, tail pinch response, righting reflex, catalepsy, proprioception, forelimb strength, visual placing response, wheelbarrowing, hopping reaction, hindlimb landing foot splay, and pupil response
